# Maladaptive coping styles moderate the relationship between information on cancer treatment and psychosocial symptoms: an Italian multicenter study

**DOI:** 10.3389/fpsyg.2024.1338193

**Published:** 2024-06-20

**Authors:** L. Zerbinati, F. Folesani, R. Caruso, M. Belvederi Murri, M.G. Nanni, S. Righetti, L. Chiefari, A. Muscettola, T. Toffanin, A. Costantini, B. Zaccagnino, F. Ruffilli, L. Grassi

**Affiliations:** ^1^Institute of Psychiatry, Department of Neuroscience and Rehabilitation, University of Ferrara, Ferrara, Italy; ^2^Psycho-oncology Service, Villa Margherita Clinic, Rome, Italy; ^3^Psycho-Oncology Service, Palliative Care, Pain Therapy and Integrative Medicine Unit, IRCCS Istituto Romagnolo per lo Studio dei Tumori (IRST) “Dino Amadori”, Meldola, Italy

**Keywords:** awareness, cancer, coping strategies, oncology, psychological distress, quality of life

## Abstract

**Objectives:**

Disclosing information on diagnosis, prognosis and treatment is a delicate process in oncology, although awareness levels have over time increased in people with cancer. However, individual characteristics should be considered when communicating difficult information. We conducted a multicentric study to explore the moderating role of coping styles on the relationship between information about cancer, quality of life and psychological distress.

**Methods:**

In the period between October 2015 and February 2016, 288 patients with a diagnosis of a solid tumor were recruited from seven Italian oncology units. All participants were administered the Distress Thermometer (DT), the Mini-Mental Adjustment to Cancer (Mini-MAC), the European Organization for Research and Treatment of Cancer (EORTC) Core Quality of Life Questionnaire (QLQ-C30), and the EORTC QLQ 25-item information module (INFO25). We explored the moderating effect of coping style with quality of life (QoL) and distress (DT) as dependent variables and information on cancer treatment as independent variable.

**Results:**

Low levels of anxious preoccupation significantly moderated the relationship between information on treatment and QoL (R2 6%, *p* < 0.001), while low and medium levels of hopelessness significantly moderated the relationship between information on treatment and DT (R2 = 14%, *p* = 0.033). Adaptive coping strategies, such as fighting spirit and fatalism, and borderline strategies such as avoidance, did not play a role in this relationship.

**Conclusion:**

Taking into account and evaluating coping mechanisms in cancer care is a priority when disclosing information on treatments, in order to tailor communication style to individual features.

## 1 Introduction

Having clear information about one’s own state of health is one of the main needs of cancer patients (Caruso et al., 2000) and an essential condition to make therapeutic decisions and set up planning of care (Weeks et al., 1998; Enzinger et al., 2015; Epstein et al., 2016).

A growing interest in the field of information given to patients with a diagnosis of cancer has spread in the last years (Wan et al., 2020; Sutar and Chaudhary, 2022), leading to a consensus on the worldwide importance of cancer diagnosis awareness.

In the past, Italy has been generally considered a country with a predominant “non-disclosure” culture [Costantini et al., 2006; AIOM (Associazione Italiana di Oncologia Medica), 2019], although cancer patients express needs for clear and complete communication (Goss et al., 2015), and details explaining the diagnosis (Truccolo et al., 2019). However, the Code of Ethics (FNOMCeO, 2014) and the most recent ‘Rules on informed consent and advance processing provisions’ (Ministero della Salute, 2017) underline the importance of awareness on the right to self-determination, the right to be fully informed about one’s own state of health, and on privacy, in order to decide with whom to share confidential information (including the family). A multicentric Italian study (Costantini et al., 2015) observed a prevalence of diagnosis awareness of 84% in cancer patients, similar to previous studies (86%) and confirming an increasing trend of information and communication compared to the past (Bracci et al., 2008; Costantini et al., 2015).

In spite of these data, doctor-patient relationship is still characterized by serious communication gaps, especially regarding prognostic and treatment information (Gianinazzi et al., 2022). In fact, while doctors and nurses generally consider communication of cancer diagnosis as mandatory, this is not always true for prognosis: 90% of doctors consider it appropriate to inform the patient of the disease, but only 54% consider adequate to be completely honest when cancer is in an advanced phase (Di Giacomo et al., 2012). Cancer treatment, such as chemotherapy represent a challenge to patients’ quality of life, contributing to somatic side-effects, poorer physical status, reduction of performance and worsening of functional dimensions, including interpersonal relationship and psychological well-being (Lewandowska et al., 2020).

While side effects of therapy have been diminishing over the years due to improvement in treatment regimens, the psychosocial implications of communicating information on treatment still remain an important and scarcely addressed issue (Turon et al., 2022; Vardy et al., 2022). For example, patients information needs are often high at the beginning of radiotherapy and education programs on how to convey a good communication have been shown to reduce psychological distress (Halkett et al., 2018). Meeting the information needs of patients, especially in the early stages of the disease, may in fact lead to greater satisfaction with care, better quality of life and more realistic expectations on cancer treatments (Larson et al., 1996; DeMartini et al., 2019; Tran et al., 2019). However, not all patients react equally to the disease and the information provided, and individual factors such as coping mechanisms influence psychosocial distress secondary to the cancer (Nipp et al., 2016).

Several studies considered coping strategies as an independent predictor of emotional distress and psychological outcomes, with some coping strategies being more adaptive than others (Dunkel-Schetter et al., 1992; Shimizu et al., 2015; Meggiolaro et al., 2016). In particular, strategies such as avoidance or passive acceptance and resignation to illness, sense of impotence and despair (hopelessness) and, overall, all those characterized by a general disengagement are associated with a worse long-term adaptation, reduced QoL and negative effects on mood, as opposed to strategies focused on commitment such as fighting spirit, problem solving and the search for social support (Lutgendorf et al., 2002; Hack and Degner, 2004; Costanzo et al., 2006).

Considering the importance of treatment adherence and psychosocial adaptation when information about cancer treatment is provided, the aim of the present study is to evaluate the role of coping strategies as moderators in the relationship between treatment information and patients’ quality of life and distress. We hypothesize that the impact of treatment information on quality of life and distress is moderated by patients’ individual coping mechanisms. This understanding could lead to improved and targeted doctor-patient communication strategies, which we plan to test in future studies.

## 2 Materials and methods

### 2.1 Participants

This study is part of an Italian multicentric study on cancer awareness, recruiting patients in different time periods from several oncology centers in Italy. Data from 262 patients were already presented in a previous paper (Costantini et al., 2015) examining levels of awareness, satisfaction with care, emotional distress and adjustment to illness. The present study extended the previous results and recruited patients between October 2015 and February 2016 from seven Italian oncology centers, namely Aviano, Milan, Bari, Cosenza, Rome, Pescara, Ascoli Piceno, Ferrara and Siracusa. It differs from the previous published paper in that it includes a different sample and focuses on the role of copying strategies and treatment awareness. The recruitment of participant took place after completion of oncologic treatment.

Inclusion criteria were: (1) solid tumor diagnosis; (2) age > 18 years; (3) diagnosis of cancer within the previous year; (4) cognitive abilities sufficiently intact to fill out questionnaires (explored through a clinical interview); (5) fluence in Italian language and no language difficulties; (6) not enrolled in other studies. Exclusion criteria were: (1) brain metastases; (2) severe cognitive and/or physical impairment. The study was approved by the regulations and ethics of the Committee for the Protection of Persons as adopted by the Local Health Trust of each center. After each patient provided his/her written consent to participate, an individual appointment was planned in the outpatient cancer service.

### 2.2 Assessments

Following the approach used in a previous study on awareness by Costantini et al. (2015), all patients in our study were administered several standardized scales by a trained psycho-oncologist. These included the Distress Thermometer (DT), the Mini-Mental Adjustment to Cancer (Mini-MAC), the European Organization for Research and Treatment of Cancer (EORTC) Core Quality of Life Questionnaire (QLQ-C30), and the EORTC QLQ 25-item information module (INFO25). Additionally, participants were asked to evaluate their subjective beliefs about the severity and curability of their disease using two visual analogical scales, rating their perception from 1 (very difficult to cure/very serious) to 10 (very easy to cure/not serious). Diagnosis awareness was further assessed through an open-ended question: ‘What is the nature of your illness and why are you being treated in hospital?’. Responses were clinically evaluated to ascertain the presence or absence of diagnosis awareness, particularly noting responses that indicated a general lack of understanding of their cancer condition, such as ‘I am in the hospital because of a backache’ or ‘swollen nodule, ‘which were categorized as showing an absence of awareness. This dichotomous evaluation was based on the direct reflection of their understanding of their cancer condition as diagnosed by their healthcare providers.

The Distress Thermometer (DT) measures the level of emotional distress over the previous week (Roth et al., 1998; Grassi et al., 2013). It has been developed by the Distress Management Guidelines panel within the National Comprehensive Cancer Network and consists of a visual analog tool asking the subject to rate his or her level of distress through a 0–10 scale (from ‘no distress’ = 0 to ‘extreme distress’ = 10). A score ≥ 4 has been considered as the most sensitive and sensible cutoff for distress (‘caseness’).

The Mini-mental Adjustment to Cancer scale (Mini-MAC) was used to assess adjustment to cancer (Watson et al., 1994; Grassi et al., 2005). It is a 29-item self-report measure devised to evaluate the patient’s coping styles, over the last 2 weeks, through five subscales: fighting spirit (4 items, score ranging between 4 and 16) measuring the tendency to confront and actively face cancer; hopelessness (8 items, score ranging between 8 and 32) measuring the tendency to adopt a pessimistic attitude about the illness; anxious preoccupation (8 items, score ranging between 8 and 32) measuring anxiety and tension about cancer; fatalism (5 items, score ranging between 5 and 40), assessing resigned and fatalistic attitudes toward the illness; and avoidance (4 items, score ranging between 4 and 16) evaluating the tendency to avoid confrontation with illness. The Mini-MAC scale utilizes a 4-point Likert scale for each item, where responses range from ‘Definitely does not apply to me’ (1) to ‘Definitely applies to me’ (4), assessing the current experiences of patients.

The EORTC QOL Questionnaire Core-30 (EORTC QLQ C-30) was administered to examine quality of life (QoL; Aaronson et al., 1993; Marzorati et al., 2019). It is a validated, widely used 30-item questionnaire examining the intensity of current possible functional problems (items 1–5), the intensity of symptoms and/or other problems in the last week (items 6–28), and the rating of health and QoL in the last week (items 29–30). The scale consists of five functional scales (physical, role, emotional, social, and cognitive function), three symptom scales (fatigue, pain, and nausea/vomiting), one global QoL scale, and six single items (symptoms and financial impact). This instrument comprises 30 items. For the first 28 questions, a four-point Likert scale is used, where 1 signifies ‘not at all’, 2 indicates ‘a little’, 3 represents ‘quite a bit’, and 4 means ‘very much’. Questions 29 and 30, however, utilize a seven-point scale that spans from 1, meaning ‘very poor’, to 7, indicating ‘excellent’. To assess quality of life of the participants, we considered the answer the question #30 of the EORTC-C30 (“How would you rate your quality of life in the previous 7 days?”) that requires an evaluation according to a liker scale from 1 (the worst) to 7 (the best).

The EORTC Quality of Life Group information questionnaire-25 items (EORTC QOL-Q INFO-25; Arraras et al., 2010) was used to measure the amount of information received on four subscales: about the disease (four items), medical tests (three items), treatment (six items), and other services (four items). Each of the 25 questions is scored on a four-point Likert scale response format (1 = not at all, 2 = a little, 3 = quite a bit, and 4 = very much), except for the dichotomous (yes/no) questions 51 and 52 and 54 and 55. For the purpose of this study the treatment subscale was used (scores ranging from 6 to 24).

### 2.3 Statistical analysis

We analyzed all data in the following order. First, Pearson correlation test was used between QoL, DT, EORTC QLQ C-30 somatic symptoms, EORTC QOL-Q INFO-25 treatment subscale and Mini MAC coping styles. Also we used in the analysis the score to the single EORTC QLQ C-30 items 29 and 30 (“How would you rate your health in the previous week?,” “How would you rate your quality of life in the previous week?”). Regarding treatment information, we used the EORTC QOL-Q INFO-25 treatment subscale items (from 38 to 43: “The medical treatment (chemotherapy, radiotherapy, surgery or other treatment modality)?,” “The expected benefit of the treatment?,” “The possible side-effects of your treatment?,” “The expected effects of the treatment on disease symptoms?,” “The effects of the treatment on social and family life?,” “The effects of the treatment on sexual activity?”).

Second, Multivariate Regression Analyses were performed with the QoL and DT as dependent variables and sociodemographic variables, performance status (Karnofsky), somatic symptoms (EORTC QLQ C-30), coping styles (Mini-MAC) and perception of curability and severity of the disease as predictors.

To explore the moderator role of coping style between information on treatment and psychosocial features, we modeled the interaction between coping style and information on treatment in predicting QoL and DT using a linear regression approach.

Descriptive and correlation analyses were conducted with IBM SPSS version 22.0. Regression analyses were run by using R vers 4.2.1. *lm package*, while *interaction* analysis was used for the creation of the figures. All tests were two-tailed with alpha set at *p* < 0.05.

## 3 Results

### 3.1 Sample characteristics

The sample characteristics are reported in Table 1. A total of 288 patients with cancer were recruited, of whom 177 females (61.7%) and 111 males (38.3%) with a mean age of 60.2 ± 12.4 years. 43.2% had a metastatic disease and 56.8% a local disease. Breast (32.8%) and gastrointestinal cancer (34.8%) were the most common diagnoses, followed by gynecologic cancer (12.2%). Almost all patients (94.4%) were aware of their diagnosis. The majority of patients reported that they did not or infrequently perceive their family as protecting them from bad news (“Never” 38.1%, “Sometimes” 31.0%) and that they did not or infrequently perceive the need to talk more about their disease with their family (“Never” 36.3%, “Sometimes” 47.0%). Mean values and SD of each coping style are reported in Table 1. Patients displayed higher tendency toward hopelessness and fighting spirit and lower toward anxious preoccupation and fatalism.

**Table 1:** Sample characteristics.

Variable	*N* = 288	
	N (M)	% (SD)
Gender, female	177	61.7
Age, mean (SD)	60.2	12.4
Education ≥13 years	164	57.5
Living alone	36	12.6
Metastatic disease	124	43.2
Karnofsky, mean (SD)	72.7	33.2
Distress thermometer, mean (SD)	4.27	2.9
Surgery	193	67.5
Chemotherapy	194	70.8
Radiotherapy	62	22.3
Days between diagnosis and questionnaires	511.39	2506.41
Type of cancer		
Respiratory tract	21	7.3
Gastrointestinal	100	34.8
Genitourinary	19	6.6
Gynecologic	35	12.2
Head and neck	8	2.8
Breast	94	32.8
Brain	2	0.7
Other	8	2.8
Psychiatric medications	29	10.1
Psychological support	76	26.5
Awareness of diagnosis	255	94.4
Perceived severity, mean (SD)	5.05	2.56
Perceived curability, mean (SD)	6.09	2.36
Perceived protection by family		
Never	102	38.1
Sometimes	83	31.0
Often	44	16.4
Always	39	14.6
Perceived need to talk more		
Never	98	36.3
Sometimes	127	47.0
Often	25	9.3
Always	20	7.4
EORTC-QLQ-C30		
QoL	60.32	21.73
Physical function	29.14	21.93
Role function	36.64	29.33
Emotional function	29.97	22.29
Cognitive function	24.14	29.99
Social function	30.59	26.92
Fatigue	42.80	23.68
Nausea	19.49	22.55
Pain	24.69	25.33
Dyspnoea	23.74	27.93
Insomnia	31.62	29.52
Loss of appetite	25.25	34.72
Constipation	22.79	28.69
Diarrhea	15.69	23.59
Economics	25.89	29.52
INFO 25		
Disease	56.75	20.76
Tests	61.68	23.37
Treatment	52.73	21.23
Other services	30.64	22.49
Other places	24.87	29.64
Self-help	41.16	32.33
Written info	49.53	50.23
Video info	59.81	49.26
Satisfaction	58.65	24.28
Need more info	52.83	50.16
Need less info	33.98	47.60
Useful info	62.69	24.32
Mini-MAC (coping style)		
Hopelessness	14.62	5.83
Fighting	16.35	3.09
Anxious preoccupation	16.87	5.44
Fatalism	11.42	2.93
Avoidance	10.87	3.56

We compared distress, quality of life and information on treatment between individuals with and without metastatic disease (Supplementary Table S1): those with non-metastatic disease received more information on treatment (*p =* 0.023, Hedges’ g 0.3) and displayed a significantly higher quality of life (*p =* 0.002, Hedges’ g 1.35).

### 3.2 Correlation analysis

Correlation analyses are reported in Supplementary Table S2. QoL significantly correlated with somatic symptoms (R ranging from −0.500 to −0.160, *p* < 0.01), information on cancer treatment (R = 0.124, *p* < 0.05), and Mini-MAC hopelessness (R = −0.198, *p* < 0.01) and anxious preoccupation (R = −0.208, *p* < 0.01) coping styles. DT significantly correlated with somatic symptoms (except for nausea, dyspnea, loss of appetite and diarrhea; R ranging from 0.167 to 0.234, *p* < 0.01), treatment information (R = −0.271, *p* < 0.01), and Mini-MAC hopelessness (R = 0.273, *p* < 0.01) and anxious preoccupation (R = 0.391, *p* < 0.01).

### 3.3 Multivariate regression analysis

The multivariate regression analysis with the QoL as dependent variable was overall statistically significant (R2 = 35%, *F*(22, 196) = 6.33, *p* < 0.001; Table 2): QoL was significantly predicted by fatigue (Beta = −0.29, *p* < 0.01), nausea (Beta = 0.13, *p* < 0.05), loss of appetite (Beta = −0.12, *p* < 0.05), diarrhea (Beta = −0.21, *p* < 0.001), and perceived severity (Beta = 1.50, *p* < 0.05).

**Table 2:** Multivariate regression analysis with quality of life as dependent variable.

Model R^2^ = 35%, *F* = 6.33, *p* < 0.01	QoL	
Predictor	Coeff.	Std. error, CI
(Intercept)	55.64***	(12.84, CI = [30.33, 80.96])
Age	0.16	(0.11, CI = [−0.06, 0.39])
Karnofsky	−0.01	(0.04, CI = [−0.09, 0.07])
Education	−0.25	(1.15, CI = [−2.52, 2.02])
Gender (female)	1.43	(2.81, CI = [−4.11, 6.97])
Disease stage (metastatic)	−2.94	(2.67, CI = [−8.21, 2.33])
C30 Fatigue	−0.29**	(0.09, CI = [−0.47, −0.12])
C30 Nausea	0.13*	(0.07, CI = [0.00, 0.26])
C30 Pain	−0.09	(0.07, CI = [−0.22, 0.05])
C30 Dyspnea	−0.07	(0.06, CI = [−0.18, 0.05])
C30 Insomnia	−0.00	(0.05, CI = [−0.10, 0.10])
C30 Loss of appetite	−0.12*	(0.06, CI = [−0.24, −0.01])
C30 Constipation	0.00	(0.05, CI = [−0.09, 0.10])
C30 Diarrhea	−0.21***	(0.06, CI = [−0.33, −0.09])
Mini MAC Fighting	0.90	(0.59, CI = [−0.27, 2.07])
Mini MAC Hopelessness	0.24	(0.32, CI = [−0.40, 0.88])
Mini MAC Anxious preoccupation	−0.41	(0.35, CI = [−1.09, 0.28])
Mini MAC Fatalism	0.32	(0.60, CI = [−0.86, 1.51])
Mini MAC Avoidant	−0.11	(0.41, CI = [−0.93, 0.71])
Perceived severity (reverse)	1.50*	(0.71, CI = [0.10, 2.91])
Perceived curability	−0.24	(0.78, CI = [−1.78, 1.29])
DT	−0.82	(0.51, CI = [−1.83, 0.20])
INFO 25 Information on treatment	0.02	(0.06, CI = [−0.11, 0.14])

Multivariate regression analysis with quality of life as dependent variable and sociodemographic variables, performance status (Karnofsky), somatic symptoms (EORTC QLQ C-30), coping styles (Mini-MAC) and perception of curability and severity of the disease as predictors. ***p < 0.001; **p < 0.01; *p < 0.05.

The multivariate regression analysis with the DT as dependent variable displayed a significant model (R2 = 29%, F(22, 196) = 5.01, *p* < 0.01; Table 3): distress was significantly predicted by female gender (Beta = 1.16, *p* < 0.01), constipation (Beta = 0.02, *p* < 0.05), anxious preoccupation coping style (Beta = 0.14, *p* < 0.01) and treatment information (Beta = −0.04, *p* < 0.001).

**Table 3:** Multivariate regression analysis with psychological distress as dependent variable.

Model R^2^ = 29%, *F* = 5.01, *p* < 0.01	DT	
Predictor	Coeff.	Std. error, CI
(Intercept)	5.94**	(1.80, CI = [2.38, 9.49])
Age	−0.01	(0.02, CI = [−0.04, 0.02])
Karnofsky	0.01	(0.01, CI = [−0.00, 0.02])
Education	−0.12	(0.16, CI = [−0.44, 0.19])
Gender (female)	1.16**	(0.38, CI = [0.41, 1.90])
Disease stage (metastatic)	−0.31	(0.37, CI = [−1.03, 0.42])
C30 Fatigue	0.01	(0.01, CI = [−0.02, 0.03])
C30 Nausea	0.00	(0.01, CI = [−0.02, 0.02])
C30 Pain	0.01	(0.01, CI = [−0.01, 0.03])
C30 Dyspnea	−0.01	(0.01, CI = [−0.02, 0.01])
C30 Insomnia	0.00	(0.01, CI = [−0.01, 0.02])
C30 Loss of appetite	−0.01	(0.01, CI = [−0.03, 0.00])
C30 Constipation	0.02*	(0.01, CI = [0.00, 0.03])
C30 Diarrhea	−0.02	(0.01, CI = [−0.03, 0.00])
Mini MAC Fighting	−0.07	(0.08, CI = [−0.23, 0.09])
Mini MAC Hopelessness	0.01	(0.04, CI = [−0.08, 0.10])
Mini MAC Anxious preoccupation	0.14**	(0.05, CI = [0.05, 0.24])
Mini MAC Fatalism	0.07	(0.08, CI = [−0.10, 0.23])
Mini MAC Avoidant	−0.07	(0.06, CI = [−0.18, 0.05])
Perceived severity (reverse)	0.02	(0.10, CI = [−0.18, 0.21])
Perceived curability	−0.09	(0.11, CI = [−0.30, 0.12])
QoL	−0.02	(0.01, CI = [−0.03, 0.00])
INFO 25 Information on treatment	−0.04***	(0.01, CI = [−0.06, −0.02])

Multivariate regression analysis with psychological distress (DT) as dependent variable and sociodemographic variables, performance status (Karnofsky), somatic symptoms (EORTC QLQ C-30), coping styles (Mini-MAC) and perception of curability and severity of the disease as predictors. ***p < 0.001; **p < 0.01; *p < 0.05.

### 3.4 Interaction analyses

**Figure 1:**
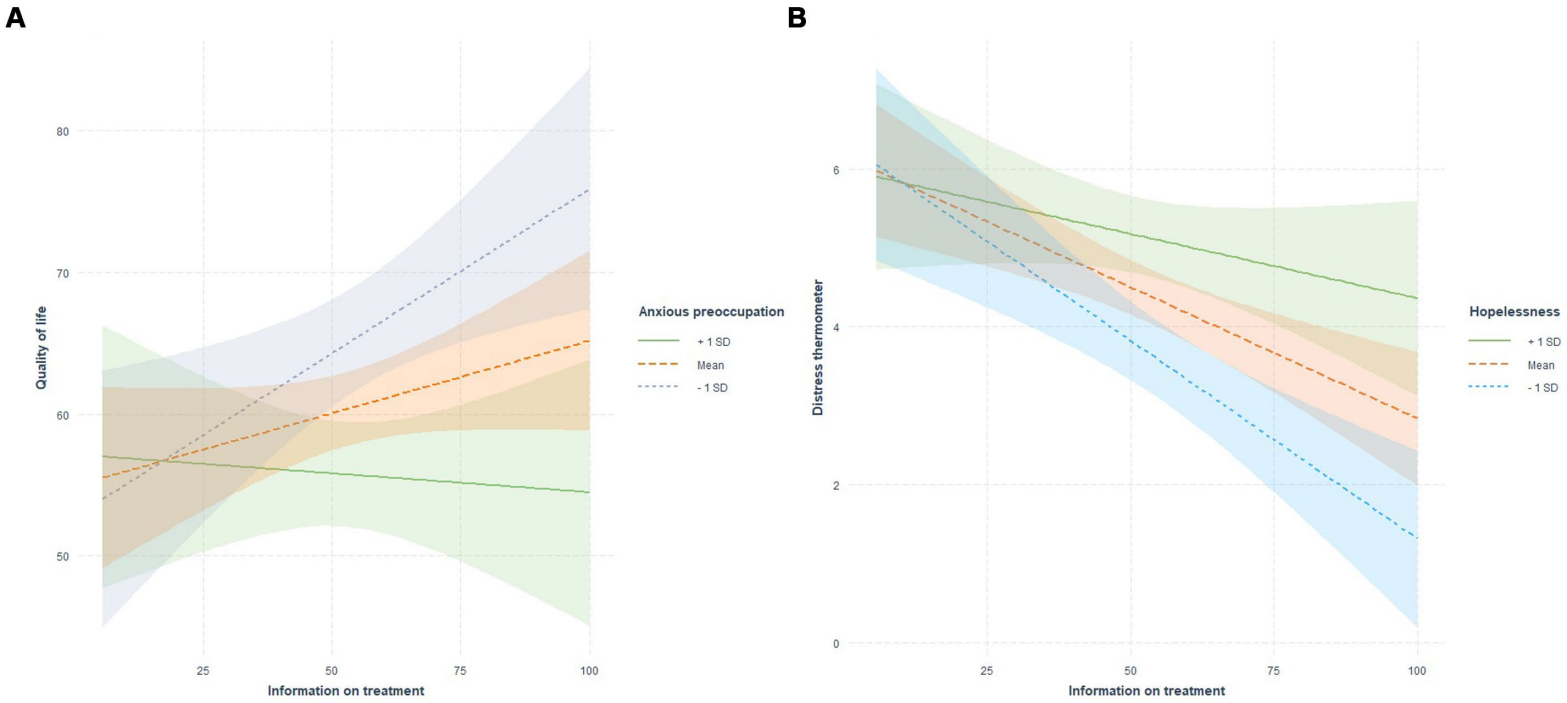
Coping styles as moderators between information on treatment and quality of life and distress.

The linear regression analyses displayed a significant interaction between anxious preoccupation and information on treatment in predicting QoL (R2 6%, *p* < 0.001; Figure 1A; Supplementary Figure S1; Supplementary Table S3). Specifically, low levels of anxious preoccupation significantly moderated the relationship between information on treatment and QoL (Supplementary Table S4).

In the model including information on treatment, hopelessness and DT, the interaction between hopelessness and treatment information significantly predicted the levels of distress (R2 = 14%, *p* = 0.033; Figure 1B; Supplementary Figure S2; Supplementary Table S5). Specifically, low and medium levels of hopelessness significantly moderated the relationship between information on treatment and DT.

No other interaction between coping styles and information on treatment displayed a significant effect on QoL or DT (Supplementary Tables S7, S8). All analyses were adjusted for multiple comparisons using the Benjamini Hochberg test (Benjamini and Hochberg, 1995).

## 4 Discussion

To our knowledge, this is the first Italian multicenter study exploring the moderating role of coping strategies in the relationship between information about cancer treatments and psychological consequences. Our study represents a significant step forward in understanding the nuanced dynamics between coping strategies, cancer treatment information, and psychological outcomes, particularly within the Italian context.

Firstly, we observed a complex interplay between coping mechanisms and psychological well-being among cancer patients. While our findings align with a prior study regarding patients’ awareness of their diagnosis and levels of hopelessness, fighting spirit, anxious preoccupation, and fatalism, a closer examination is warranted to discern potential cultural or methodological nuances (Grassi et al., 2005).

The predictive role of somatic symptoms in determining quality of life and psychological distress underscores the multifaceted nature of cancer adaptation. Psychological distress was mainly predicted by female gender, the somatic symptom “constipation,” anxious preoccupation and information on treatment. Moreover, individuals with metastatic disease displayed lower levels of quality of life and receive less information on treatment. This is in line with the available literature when explored according to gender (Linden et al., 2012; Parás-Bravo et al., 2020), copying styles (Obispo et al., 2023) and constipation (Wickham, 2017), and metastatic vs. non-metastatic disease on quality of life (Zheng et al., 2023). As for the lower amount of information on treatment received by those with metastatic disease, this could be explained by a tendency of physician to non-disclosure in conditions with poor prognosis (Fumis et al., 2012). However, a critical appraisal of potential confounders and mediators is crucial to elucidate the underlying mechanisms driving these associations and to inform targeted interventions aimed at improving patient outcomes.

Furthermore, our study sheds light on the moderating effects of coping strategies, particularly anxious preoccupation and hopelessness, in shaping patient responses to treatment information. When examining the role of coping, we found that lower levels of anxious preoccupation were associated with better quality of life when individuals were given more information on treatment. Also, higher levels of anxious preoccupation were negatively related to quality of life in the presence of more information on treatment, although the correlation did not reach statistical significance, probably because of the limited sample size. Low and medium levels of hopelessness were associated with significantly lower levels of psychological distress when patients were given more information on treatment.

These findings corroborate existing literature on the detrimental impact of maladaptive coping strategies on cancer patient outcomes, emphasizing the need for personalized interventions tailored to individual coping styles, by further exploring their moderating role when receiving information on treatment (Nipp et al., 2016). These coping strategies, have been shown to have a negative influence on physical and mental quality of life in cancer patients during chemotherapy (Lauriola and Tomai, 2019), as well as on doctor-patient relationship (Meggiolaro et al., 2016). Coping mechanisms and personality features shape the adjustment to cancer and thus the reaction to information received (You et al., 2018): neuroticism and “avoidance coping strategies” (e.g., avoidance and denial) are associated with negative affect in the adjustment to the disease, while “active coping strategies” (e.g., fighting spirit and support seeking) are associated with prominent traits of extraversion and neuroticism.

Interestingly, our study delineates distinct moderation effects of anxious preoccupation and hopelessness on quality of life and psychological distress, respectively. These two coping strategies are frequently considered linked together (Cho et al., 2020) and were moderators of two distinct constructs, namely QoL and distress. Since QoL in our model is predicted by physical symptoms, it is possible that the moderation effect of anxious preoccupation is explained by a tendency toward worries and complaints about physical health and a tendency to focus attention to somatic symptoms. On the other hand, the moderating effect of hopelessness on DT might be attributed to a tendency toward demoralization, which is typically characterized by psychological distress and hopelessness features (Clarke and Kissane, 2002; Grassi and Nanni, 2016). These observations confirm what reported in a previous study on breast cancer patients, highlighting the association of health anxiety with anxious preoccupation, and hopelessness with demoralization (Grassi et al., 2004).

On the other side, more adaptive coping styles examined in the study (i.e., fatalism, fighting spirit and avoidance) did not display any interaction effect with the amount of information about treatment. Therefore, hopelessness and anxious preoccupation are confirmed to act as maladaptive coping strategies, associated with higher emotional distress and lower quality of life (Grassi et al., 2005; Seok et al., 2013; Meggiolaro et al., 2016; Cheng et al., 2019), while fatalism and fighting spirit are confirmed to be related to adjustment to cancer (Grassi et al., 2005; Czerw et al., 2015; Lauriola and Tomai, 2019).

*Limitations*. There are limitations to be considered when interpreting the results of the present study. First, the research was conducted on a nation-wide level. Even though the sample size was fairly large, we could not exclude possible cultural and regional influences in adaption mechanisms to cancer, precluding generalizability of our results. Moreover, due to the multicentric nature of the study, the patients were informed by many different doctors, who probably adopt a different information style. Further studies should extend this research by including a greater sample size and more areas of Italy, to confirm the results and extend its generalizability by taking into account regional differences. Second, in this study we did not consider differences related to socio-demographic and clinical characteristics, while it has been reported that emotional distress varies with age, gender, disease site/stage, education and income, furtherly highlighting the need of a personalized approach to cancer care (Harms et al., 2019; Lewis et al., 2021; Brauer et al., 2022; Shafiq et al., 2022; Sutton et al., 2022). Finally, although we evaluated diagnosis awareness, we did not evaluate prognosis awareness among our study participants. Understanding patients’ awareness of their prognosis is crucial, especially in the setting of advanced cancer, where treatment expectations can significantly influence decision-making processes and outcomes. The absence of this evaluation might limit the applicability of our findings to scenarios where prognosis awareness directly impacts patient choices and care outcomes because of unrealistic expectations. Future studies should consider incorporating an assessment of prognosis awareness to provide a more comprehensive understanding of its influence on patient expectations and treatment decisions in advanced disease stages.

*Clinical implications*. Communication skills are crucial in doctor-patient relationship, influencing adherence to treatment and participation to the process of care (Mills and Sullivan, 1999; Haskard Zolnierek and DiMatteo, 2009). As reported in several communication guidelines, it is necessary for clinicians to discuss treatment options in a way that preserves patients’ hope, promotes autonomy, and facilitates understanding (Gilligan et al., 2017). These goals are feasible if the clinician does not overlook the characteristics of the person to whom information are given, adapting the amount and type of information both to the abilities to cope and to the health literacy levels of the individual (Halbach et al., 2016). Previous attempts were also made to provide communication guidelines for different personality types (Kallergis, 2008). Our study contributes valuable insights into the complex interplay between coping strategies, treatment information, and psychological outcomes among cancer patients. Moving forward, a critical synthesis of existing literature and continued research efforts will be crucial in advancing our understanding of cancer adaptation mechanisms and informing targeted interventions aimed at improving patient well-being, further advance our comprehension of these complex processes and effectively inform clinical practice.

## 5 Conclusion

In conclusion, our study underscores the pivotal role of tailoring communication in healthcare settings, particularly regarding information on cancer treatments and their psychological ramifications. The findings reveal a nuanced interplay between coping strategies and the quantity of treatment-related information, shedding light on their differential impacts on quality of life and psychological distress among cancer patients. While the majority of patients demonstrated adequate awareness of their diagnosis, our analysis identified significant predictors of both quality of life and psychological distress. Notably, somatic symptoms emerged as primary determinants of quality of life, underscoring the importance of addressing physical manifestations alongside psychological well-being in cancer care. Moreover, our study highlights the moderating influence of coping strategies, particularly anxious preoccupation and hopelessness, in shaping patient outcomes in the context of treatment information. These findings emphasize the need for personalized approaches to communication in healthcare delivery, recognizing individual coping styles and psychological states. Clinicians must navigate a delicate balance, fostering hope, autonomy, and understanding while tailoring the dissemination of information to patients’ coping abilities and health literacy levels. Moreover, routine monitoring of psychological distress in outpatient settings is paramount for ensuring holistic patient care. Moving forward, future research should seek to validate and extend these findings across diverse cultural contexts and patient populations, addressing potential regional influences and socio-demographic disparities. Additionally, efforts to develop communication guidelines tailored to different personality types hold promise for optimizing patient-provider interactions and enhancing overall cancer care outcomes. By prioritizing patient-centered communication strategies, healthcare professionals can empower individuals facing cancer with the support and information needed to navigate their journey toward healing and resilience.

## Data availability statement

The raw data supporting the conclusions of this article will be made available by the authors, without undue reservation.

## Ethics statement

The studies involving humans received approval from the Ethical Committees of the Sant’Anna Hospital, Azienda Unità Sanitaria Locale (AUSL) of Ferrara, Ferrara and Sant’Andrea University Hospital, Rome (Costantini et al., 2015). The studies were conducted in accordance with the local legislation and institutional requirements. The participants provided their written informed consent to participate in this study.

## Author contributions

LZ: Formal analysis, Writing – original draft. FF: Formal analysis, Writing – original draft. RC: Writing – review & editing. MB: Writing – review & editing. MN: Writing – review & editing. SR: Writing – original draft. LC: Writing – original draft. AM: Writing – original draft. TT: Writing – review & editing. AC: Investigation, Writing – review & editing. BZ: Investigation, Writing – review & editing. FR: Investigation, Writing – review & editing. LG: Supervision, Writing – review & editing.

## Group members of the Italian society of psycho-oncology (SIPO) quality of life in cancer working group

The Italian society of psycho-oncology (SIPO) quality of life in cancer working group consists of Paolo Marchetti, Serena Brunetti, Mauro Carone, Valentina Padolecchia, Annarita Di Silvestre, Paolo Tralongo, Donatella Morale, Maria Antonietta Annunziata, Angela Piattelli, Salvatore Palazzo, Sabrina Marini, and Maria Domenica Iuvaro.
