# Supplementary material for: Maladaptive coping styles moderate the relationship between information on cancer treatment and psychosocial symptoms: an Italian multicenter study
Source: Front Psychol. 2024 Jun 20;15:1338193. doi: 10.3389/fpsyg.2024.1338193 (PMC11223643; doi:10.3389/fpsyg.2024.1338193)
Supplement: Supplementary file 1 [file Data_Sheet_1.PDF]

SUPPLEMENTARY MATERIAL

Results

Table S1. Mean comparison between metastatic and non-metastatic disorder

**Variable**	Metastatic		Non metastatic		*p*	Hedges' g
	N	M (SD)	N	M (SD)		
Distress thermometer	87	4.17 (2.85)	168	4.29 (2.93)	0.756	0.041
Info treatment	88	2.46 (0.60)	169	2.65 (0.65)	0.023*	0.300
QoL	97	54.90 (22.69)	174	63.31 (20.70)	0.002*	1.350

Table S2. Correlations

	QOL	DT
QoL	1	**-.228****
DT	**-.228****	1
Fatigue	**-.500****	**.234****
Nausea	**-.242****	.123
Pain	**-.461****	**.220****
Dyspnea	**-.358****	.056
Insomnia	**-.283****	**.167****
Loss appetite	**-.374****	.123
Constipation	**-.160****	**.211****
Diarrhea	**-.270****	-.082
Economic status	**-.242****	.074
INFO25 Info on treatment	**.124***	**-.271****
Mini MAC Hopelessness	**-.198****	**.273****
Mini MAC Fighting	.092	-.035
Mini MAC Anxious preoccupation	**-.208****	**.391****
Mini MAC Fatalism	-.033	.120
Mini MAC Avoidance	.033	.041

* $p < 0.05$; ** $p < 0.01$

Figure S1. Moderator analysis of anxious preoccupation coping between information on treatment and quality of life

ANXIOUS PREOCCUPATION

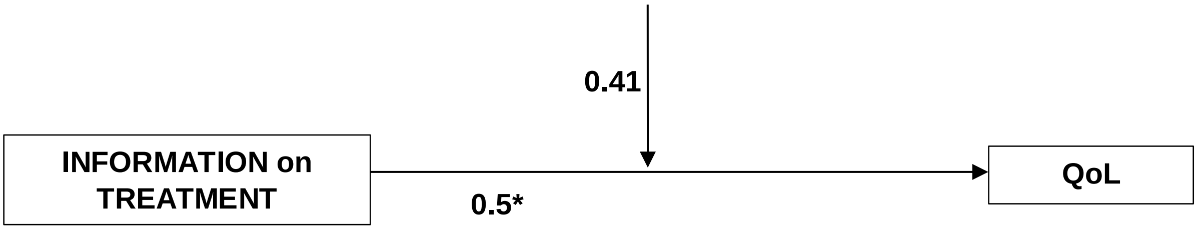

Model R2 6%, X*W – 0.024, *p* = 0.0422*

Table S3. Analysis of the interaction between anxious preoccupation and information on treatment on QoL

<table>
<tr><th>Model R2 = 6%, F = 6.996, p < 0.001</th><th>Estimate</th><th>Std. Error</th><th>t value</th><th>Pr(>|t|)</th></tr>
<tr><td>(Intercepts)</td><td>48.08569</td><td>11.82085</td><td>4.068</td><td>6.37e-05 ***</td></tr>
<tr><td>Anxious preoccupation</td><td>0.40603</td><td>0.67369</td><td>0.603</td><td>0.5473</td></tr>
<tr><td>Information on treatment</td><td>0.50345</td><td>0.20153</td><td>2.498</td><td>0.0131 *</td></tr>
<tr><td>Anxious preoccupation:Information on treatment</td><td>-0.02374</td><td>0.01163</td><td>-2.042</td><td>0.0422 *</td></tr>
</table>

*** p < 0.001; ** p < 0.01; * p < 0.05

Table S4. Anxious preoccupation levels and interaction with information on treatment

	**Estimate**	**Std Error, CI**	***p***
AP = 11.46	0.23 **	0.09, CI = 0.06, 0.40	0.01
AP= 16.89	0.10	0.06, CI = -0.02, 0.23	0.10
AP = 22.33	-0.03	0.09, CI = -0.21, 0.15	0.77

*** p < 0.001; ** p < 0.01; * p < 0.05

AP : Anxious preoccupation

Figure S2. Moderator analysis of hopelessness coping between information on treatment and distress

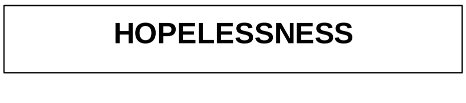

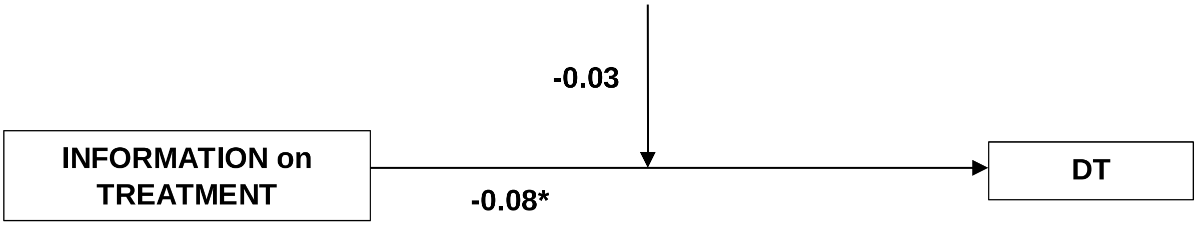

Model R2 14%, X*W 0.003, *p* = 0.04

Table S5. Analysis of the interaction between hopelessness and information on treatment on DT

**Model R2 = 14%, F = 14.2, *p* = 0.026**	Estimate	Std. Error	t value	Pr(>|t|)
(Intercepts)	6.586976	1.282391	5.136	5.93e-07 *
Hopelessness	-0.029546	0.082152	-0.360	0.719437
Information on treatment	-0.075454	0.021702	-3.477	0.000606 ***
Hopelessness:Information on treatment	0.002931	0.001417	2.069	0.039655 *

*** p < 0.001; ** p < 0.01; * p < 0.05

Table S6. Hopelessness levels and interaction with information on treatment on DT

	**Estimate**	**Std Error, CI**	***p***
H = 8.63	-0.05 ***	0.01, CI = -0.07, -0.03	0.00
H = 14.41	-0.03 ***	0.01, CI = -0.05, -0.02	0.00
H = 20.19	-0.02	0.01, CI = -0.04, 0.01	0.17

*** p < 0.001; ** p < 0.01; * p < 0.05.

H : hopelessness

Table S7. Models of the interaction between coping styles and information on treatment on QoL

	Estimate	Std. Error	t value	Pr(>|t|)
**Hopelessness**<br>**Model R2 = 5%, F = 5.682, *p* < 0.001**				

(Intercepts)	51.37678	10.10685	5.083	7.29e-07 ***
Hopelessness	0.20501	0.64385	0.318	0.7504
Information on treatment	0.36178	0.17020	2.126	0.0345 *
Hopelessness:Information on treatment	-0.01751	0.01106	-1.584	0.1146
**Fighting**				
**Model R2 = 2%, F = 2.339, *p* = 0.07408**				
(Intercepts)	62.46542	19.04557	3.280	0.00119 **
Fighting	-0.55051	1.16426	-0.473	0.63674
Information on treatment	-0.19981	0.33067	-0.604	0.54622
Fighting:Information on treatment	0.02002	0.01982	1.011	0.31321
**Fatalism**				
**Model R2 = 0.1%, F = 1.554, *p* = 0.2012**				
(Intercepts)	63.687022	14.882917	4.279	2.66e-05 ***
Fatalism	-0.896474	1.263303	-0.710	0.479
Information on treatment	0.015723	0.258548	0.061	0.952
Fatalism:Information on treatment	0.009768	0.021774	0.449	0.654
**Avoidant**				
**Model R2 = 1%, F = 1.91, *p* = 0.1284**				
(Intercepts)	38.47078	11.31149	3.401	0.00078 ***
Avoidant	1.43501	1.02022	1.407	0.16079
Information on treatment	0.37372	0.19344	1.932	0.05448
Avoidant:Information on treatment	-0.02360	0.01709	-1.381	0.16845

Signif. codes: 0 '***' 0.001 '**' 0.01 '*' 0.05 '.' 0.1 ' ' 1

Table S8. Models of the interaction between coping styles and information on treatment on DT

	Estimate	Std. Error	t value	Pr(>|t|)
**Anxious preoccupation**				

**Model R2 = 21%, F = 21.21, *p* = 0.004**				
(Intercepts)	51.37678	10.10685	5.083	7.29e-07 ***
Hopelessness	0.20501	0.64385	0.318	0.7504
Information on treatment	0.36178	0.17020	2.126	0.0345 *
Hopelessness:Information on treatment	-0.01751	0.01106	-1.584	0.1146
**Fighting**				
**Model R2 = 8%, F = 7.771, *p* = 0.169**				
(Intercepts)	2.855741	2.473948	1.154	0.250
Fighting	0.220033	0.151168	1.456	0.147
Information on treatment	0.025800	0.042997	0.600	0.549
Fighting:Information on treatment	-0.004005	0.002578	-1.554	0.122
**Fatalism**				
**Model R2 = 8%, F = 8.163, *p* = 0.002**				
(Intercepts)	3.680855	1.900557	1.937	0.054
Fatalism	0.234956	0.162362	1.447	0.149
Information on treatment	-0.016519	0.033125	-0.499	0.618
Fatalism:Information on treatment	-0.001839	0.002814	-0.654	0.514
**Avoidant**				
**Model R2 = 7%, F = 6.56, *p* < 0.001**				
(Intercepts)	7.027569	1.505488	4.668	5.15e-06 ***
Avoidant	-0.064460	0.135841	-0.475	0.6356
Information on treatment	-0.057478	0.025725	-2.234	0.0264 *
Avoidant:Information on treatment	0.001893	0.002278	0.831	0.4067

Signif. codes: 0 '***' 0.001 '**' 0.01 '*' 0.05 '.' 0.1 ' ' 1
